# ASSOCIATIONS BETWEEN THE GERIATRIC DEPRESSION SCALE AND APNEA-HYPOPNEA INDEX IN OLDER ADULTS

**DOI:** 10.1093/geroni/igad104.3198

**Published:** 2023-12-21

**Authors:** Alyssa Persad, Edward Hammel, Mitchell Roberts, Erica Sappington, Paul Dagum, Jeffrey Iliff, Swati Rane Levendovszky, Carla VandeWeerd

**Affiliations:** UF Health Precision Health Research Center, The Villages, Florida, United States; UF Health Precision Health Research Center, The Villages, Florida, United States; UF Health Precision Health Research Center, The Villages, Florida, United States; UF Health Precision Health Research Center, The Villages, Florida, United States; Applied Cognition Inc, Redwood City, California, United States; University of Washington, Seattle, Washington, United States; University of Washington Medical Center, Seattle, Washington, United States; UF Health Precision Health Research Center, The Villages, Florida, United States

## Abstract

The prevalence of sleep apnea increases with age and depression. Sleep plays an important role in memory consolidation, therefore, changes in sleep quality may play a contributing factor to neurodegenerative diseases such as Alzheimer’s. Findings from a retrospective analysis of a randomized control sleep study are presented for healthy participants (N=33) with no self-reported mental health conditions between 11/7/2022-6/7/2023. As part of a larger study, this sub-study occurred in The Villages in a partnership between the UFHealth PHRC and Applied Cognition. Participants completed the Geriatric Depression Scale (GDS-15), and Polysomnography (PSG), with the Apnea-Hypopnea Index (AHI) extracted from the PSG data. The relationship between sleep apnea and depression was examined using descriptive statistics and linear regression. The mean age of participants was 61.9, with 55% being male. GDS-15 scores showed a strong negative correlation with AHI (p<.001), indicating that as GDS scores increased, AHI decreased. The R2 value (0.06) indicates little variation in the data and is a good fit for the model. Limited data due to the small sample size may contribute to the observed relationship. Although the effect size was small, symptoms related to depression could contribute to decreased AHI. A sample from The Villages may be biased towards a higher social cohesion lifestyle than other communities, showing how elements of depression may differently impact sleep apnea. Further studies are needed to identify further associations and implement appropriate interventions.

